# Cultivation of *Spirulina* (*Limnospira platensis*) using NPK fertilizer as a low-cost media and its implications for the semiarid regions of Southern Africa

**DOI:** 10.3389/fvets.2026.1798140

**Published:** 2026-05-19

**Authors:** Keletso Masisi, Lesedi Lebogang, Amare Gessesse

**Affiliations:** Department of Biological Sciences and Biotechnology, School of Life Sciences, Botswana International University of Science and Technology, Palapye, Botswana

**Keywords:** feed production, *Limnospira platensis*, low-cost media, protein, *Spirulina*

## Abstract

**Introduction:**

In the semi-arid regions of Southern Africa, livestock farming is a key source of livelihood, but its productivity is constrained by the availability of nutritionally adequate feed, a challenge exacerbated by climate change. The microalga *Limnospira platensis* (commonly known as *Spirulina*) presents a climate-smart alternative for producing good quality animal feed. However, its widespread use is limited by high production costs, particularly those associated with labour and cultivation media.

**Methods:**

In this study, *Spirulina* CH22 was isolated from an alkaline soda lake in the African Rift Valley and cultivated using NPK fertilizer as a low-cost growth medium. Two formulations were evaluated: NPK alone (medium T1) and NPK supplemented with iron, magnesium, and calcium (medium T7). Zarrouk medium was used as the control. Biomass production, protein content, pigment concentration, growth rate, and cost efficiency were assessed.

**Results:**

Cultivation in medium T1 resulted in lower biomass, protein, phycocyanin, and chlorophyll compared to the control. In contrast, supplementated media (medium T7) improved growth performance, yielding a 31% increase in biomass relative to Zarrouk medium, while maintaining comparable protein content (54% vs. 56%). Medium T7 also achieved a higher specific growth rate and shorter doubling time. However, chlorophyll and phycocyanin content were reduced by 25% and 27%, respectively, compared to the control. Cost analysis showed that medium T7 reduced growth medium costs by 63% based on local ingredient prices in Botswana.

**Discussion:**

These findings demonstrate that NPK-based media supplemented with key micronutrients can significantly reduce production costs while maintaining high biomass productivity of *Spirulina*. Therefore, the use of medium T7 for the cultivation of Spirulina in Southern Africa could help to significantly lower production cost and encourage its wider use as food and animal feed source upon further nutritional and safety analysis.

## 1 Introduction

In the semiarid regions of Southern Africa livestock production is an important agricultural activity supporting the livelihoods of rural communities (1, 2). Availability of feed with the right quantity and nutritional quality is critical in determining livestock productivity (2). However, availability of feed in the region suffers from the impacts of climate change which affects livestock productivity (2, 3). Some of the impacts of climate change on animal feed production include drought, soil degradation during extended dry seasons, and weather variabilities that affect plant growth (4). In addition, different studies reported spatial differences in key soil nutrients in the region such as phosphorus, nitrogen, potassium and some micronutrients which can adversely affect feed production (2, 5). Therefore, to ensure food security and protect the livelihoods of people in the region, it is important to adapt to climate change by implementing climate smart approaches for food and feed production (6). In this respect, the microalgae commonly known as *Spirulina* (*Limnospira platensis* formerly known as *Arthrospira platensis*) emerges as a climate smart and nutritive alternative source of animal feed (7, 8). *Spirulina* cultivation is considered climate smart because, unlike other crops, it is not susceptible to extreme weather conditions caused by climate change and does not require access to arable land and freshwater (6). This is especially important for the Southern African region which is prone to drought and prolonged ‘hot’ seasons (2, 9).

In addition to *Spirulina*, other microalgae such as *Chlorella*, *Schizochytrium* and *Dunaliella* have been widely studied as important sources of animal feed (10). However, due to its high protein content which ranges from 50 to 70% and its well-balanced amino acid profile, *Spirulina* is the most preferred species as a source of food and animal feed. *Spirulina* also contains vitamins, essential fatty acids, antioxidants, beta carotene and other bioactive pigments making it an excellent candidate for use as feed supplement (11–13). Thus, the use of *Spirulina* as an animal feed supplement was reported to lead to significant improvements in animal health and productivity (14–16). For example, partial replacement of fish meal with *Spirulina* in aquaculture feed helped to improve growth and animal health (17–19). Similarly, when used as poultry feed supplement, *Spirulina* not only improved animal health and growth but also enhanced the nutritional quality of egg and meat products (15, 16, 20). Feeding trials using other animals have also demonstrated improvement in animal health and productivity (21, 22). Moreover, compared to other microalgae, *Spirulina* grows well under alkaline conditions (pH 9–11) and in the presence of high salt concentration. Growth under these conditions could prevent growth of other wild microalgae making large scale cultivation free of contamination (23).

The abundance of sunlight, a warm temperature during most parts of the year, and availability of vast tracts of land that is not used for crop cultivation makes the semi-arid region of Southern Africa suitable for the cultivation of *Spirulina* that can be used as animal feed upon further feeding trials and evaluations (24). Considering that several studies have suggested the nutritional benefits and importance of low cost cultivation of *Spirulina*, its cultivation in the region, especially by reducing would be beneficial (25–28). Since animal feed accounts for up to 70% of the total production cost (29), development of cheaper and climate smart alternatives is expected to play an important role in reducing cost. In regions where livestock production is a major agricultural activity and access to quality feed is a challenge, such as Southern Africa, availability of *Spirulina* biomass with affordable price could reduce dependence on conventional feed ingredients. In addition, large-scale *Spirulina* cultivation could create new jobs and help alleviate the challenges of unemployment currently rampant in the region (30–33).

However, currently large-scale *Spirulina* cultivation in the region and thus its use as a source of food and animal feed is limited. The lack of investment on microalgae research and development in the region and absence of a critical mass of trained manpower might explain the current low emphasis on the use of *Spirulina* for feed application. High production cost associated with *Spirulina* cultivation is also a major factor limiting large-scale *Spirulina* cultivation in the region. In the cultivation of *Spirulina*, the costs of labor and growth media were identified as the two major contributors of the high production cost (25, 26). Zarrouk’s medium, which contains nitrate, bicarbonate, phosphate, sulfate, and a range of trace metal ions, is the most widely used standard medium for the cultivation of *Spirulina*. Among its components, nitrate is the major cost contributor significantly increasing production expenses and limiting the economic feasibility of large-scale cultivation. To address this challenge, several studies were carried out where nitrate in Zarrouk’s medium was replaced by other lower-cost nitrogen sources (27, 28).

Some of the low-cost nitrogen sources used for *Spirulina* cultivation replacing nitrate include ammonium salts, urea, muriate of potash, super phosphate and organic nitrogen from different waste effluents and all were reported to support growth (23, 26, 27). Of these, the use of organic nitrogen from waste effluents is the cheapest. However, using waste effluents could also raise serious safety concerns if the final product is intended for food and animal feed applications. On the other hand, the use of urea and ammonium salts for *Spirulina* cultivation poses different challenges, especially in rural areas. This is because, to prepare the growth medium, in addition to the above nitrogen sources, there is a need to add other nutrients such as sulfate, phosphate, and different metal ions. But in most rural areas these chemicals are not readily available. Another nutrient used for *Spirulina* cultivation is NPK fertilizer which was shown to support the growth of *Spirulina* and other strains such as *Arthrospira fusiformis* (23, 26, 28). Low-cost media based on locally available nutrient sources have also been successfully used for the cultivation of other microalgal species. For example media based on agricultural fertilizers and waste-derived media have been successfully used for the cultivation of *Chlorella vulgaris* leading to a reduction in the cost of cultivation while resulting in high biomass productivity (34, 35). Similarly, low-cost nutrient media were used for the cultivation of *Chaetoceros gracilis* (36) and *Scenedesmus obliquus* (37, 38) resulting in a better growth than the conventional media. These observations have thus led to the suggestion of using such low cost media for large scale production of the strains (34, 36).

One major advantage of using NPK fertilizer for *Spirulina* cultivation is that it is cheap and readily available in rural areas because it is used by farmers for crop cultivation. NPK fertilizer is used to supplement nitrogen, phosphorus, and potassium in agricultural soil. When used on plants they acquire other micronutrients such as magnesium, calcium, and iron from the soil. However when NPK fertilizer is used for *Spirulina* cultivation in liquid culture, there is no direct contact with soil and this results in deficiency of essential nutrient which in turn affect growth and biomass yield. Moreover, under the alkaline condition which *Spirulina* normally grows, urea and ammonium salts used in the formulation of NPK fertilizer are converted to ammonia which, at high concentration, inhibit growth and reduce biomass yield. Therefore, to effectively use NPK fertilizer for *Spirulina* cultivation it is important to provide the right balance of nutrients required for growth and to optimize the cultivation condition to minimize the inhibitory effects of high ammonia concentration on growth and maximum biomass yield. The aim of this study was to evaluate the growth of *Spirulina* CH22 using NPK fertilizer as a low-cost medium, determine the optimum fertilizer concentration for maximum growth, assess the effects of metal ion supplementation on biomass yield, protein content and pigment accumulation then compare the cost of the fertilizer-based media with that of Zarrouk’s medium.

## 2 Materials and methods

### 2.1 Isolation and identification of the strain

Surface water was collected from Lake Chitu, Ethiopia using sterile tubes and brought to the laboratory. Ten ml of the sample was inoculated into 90 mL sterile Zarrouk’s medium supplemented with micronutrient solution (Table 1). The culture was incubated at 30 °C with 12 h/12 h light and dark cycles using a lamp at an intensity of 20 μmol m^−2^ s^−1^, 10kLux (Handheld Photosynthesis System-Cl-340, Camas, United States) with repeated daily shaking to mix the culture. After 12 days of cultivation, 10 mL of culture was harvested at the logarithmic phase (OD₆₈₀ ≈ 0.6) was aseptically transferred to a 90 mL fresh medium and incubated as before in 250 mL flasks. After three passages a sample of the culture was serially diluted and spread on agar plates prepared by adding 15 g/L agar to Zarrouk’s medium containing 1% sodium carbonate. Sodium carbonate was sterilized separately and added to the rest of the medium after cooling. After inoculation the plates were incubated at 30 °C with 12 h/12 h light/dark cycles until colonies were visible. Individual colonies were picked and purified through repeated streaking. One isolate designated as CH22 was selected for further study.

**Table 1:** Composition of the liquid media used for cultivation of *Spirulina* CH22.

Medium component	Amount (g/l)		
	Zarrouk’s medium	Medium T1	Medium T7
NaNO_3_	2.5	–	–
K_2_HPO_4_	0.5	–	–
K_2_SO_4_	1	–	–
NaCl	1	1	1
MgSO_4_.7H_2_O	0.2	–	0.2
CaCl_2_.2H_2_O	0.04	–	0.04
FeSO_4_.7H_2_0	0.01	–	0.01
EDTA	0.08	–	0.08
NaHCO_3_	6.8	6.8	6.8
Na_2_CO_3_	10	10	10
NPK (2:3:2)	–	1	1
Trace elements*	1 mL	1 ml	1 ml

*Micronutrient solution composition (g/L): H_3_BO_3_, 2.86; MnCl_2_.4H_2_O, 1.81; ZnSO_4_.4H_2_O, 0222; NaMoO_4_.2H_2_O, 0.0177; CuSO_4_.5H_2_O, 0.08.

Genomic DNA of the selected isolate was extracted using ZR Soil Microbe DNA kit™ (Zymo Research, Orange, CA, United States) following the procedure recommended by the manufacturer and the 16S rRNA gene was amplified using 902R and 1492R primers. Additionally, the phycocyanin alpha and beta subunit genes, *cpcA and cpcB*, were also amplified using the cpcA and cpcB primers and sequenced. Identification was carried out by comparing the sequences within the National Center for Biotechnology Information (NCBI)’s database using the Basic Local Alignment Search Tool (BLAST) search.

### 2.2 Cultivation conditions

The fertilizer-based cultivation medium was formulated using a commercially available NPK fertilizer obtained locally. To determine the optimal fertilizer concentration and its effect on *Spirulina* growth, a range of NPK concentrations (0.5–5.0 g/L) was evaluated using a completely randomized design (CRD). Fertilizer concentrations were varied in increments of 0.5 g L^−1^, and biomass production was assessed to identify the concentration that supported maximum growth. Based on these preliminary experiments, 1 g/L, which yielded the highest biomass production, was selected as the optimal concentration and used as the base fertilizer medium (medium T1) for subsequent experiments. The base fertilizer medium (medium T1) was prepared by combining NPK fertilizer with NaCl, Na₂CO₃, and NaHCO₃, following the general approach described by Michael et al. (26) and Madkour and Nasr (27). To address potential micronutrient limitations associated with fertilizer-only formulations, the optimized base medium (medium T1) was supplemented with (g/l): 0.2 MgSO₄·7H₂O; 0.04 CaCl₂·2H₂O; 0.01 FeSO₄·7H₂O; and 0.08 EDTA. This supplemented formulation was designated as medium T7 and was included to evaluate the effect of additional micronutrients on growth performance and biochemical composition. Therefore, the final experimental comparison was conducted using three media: standard Zarrouk’s medium (control), the fertilizer-only medium (medium T1), and the mineral-supplemented fertilizer medium (medium T7). The compositions of all media used in this study are summarized in Table 1.

### 2.3 Measurement of growth and biomass production

Growth was determined by measuring optical density at 680 nm every other day. This was done by using 2 mL of culture and measuring the optical density using fresh media as the blank. Specific growth rate (*μ*) and doubling time (td) were calculated from optical density measurements (OD₆₈₀) obtained during the exponential growth phase of the cultures. The specific growth rate was determined from the natural logarithmic increase in optical density over time using the equation shown below and the doubling time was subsequently derived from the calculated growth rate.


$$\mu \left(day^{-1}\right)=\frac{\ln\frac{N_{t}}{N_{0}}}{T_{t}-T_{0}}$$

Where *μ* is the specific growth rate, *N*_0_ and *N*_t_ represent the initial and final cell densities, respectively, and *T*_0_ and *T*_1_ represents the initial and final cultivation times, respectively.

Doubling time of the culture in the different media was calculated as shown below.


$$td\left(day^{-1}\right)=\frac{\ln2}{\mu }$$

Where *μ* is the specific growth rate and td is the doubling time/day.

Biomass production was determined by measuring cell dry weight. After proper shaking of culture flaks, 2 mL culture was filtered using a pre-weighed glass fiber filter, washed twice using distilled water and dried at 40 °C to constant weight. Dry weight was determined using the equation below.


$$Biomass\;(g/L)=\frac{Wf-Wi}{V}$$

Where Wf is the final weight of filter paper after drying with biomass, Wi is the initial weight of the filter paper without biomass and *V* is the volume of the culture medium.

### 2.4 Determination of total protein

Total protein was determined following the Lowry method (39) as modified by Markou et al. (40). Briefly, dry *Spirulina* biomass was ground into powder and suspended in 0.1 M NaOH solution followed by sonication for 30 min using a sonicator (Model 704, Scientech Ultrasonic Cleaner, South Africa). The mixture was vortexed for 30 s, centrifuged at 6000 rpm for 5 min, and the supernatant used to measure protein content using bovine serum albumin (BSA) as a standard.

### 2.5 Measurement of chlorophyll content

To determine chlorophyll content 2 mL culture was centrifuged at 4000 rpm for 12 min and the pellet was washed three times by resuspending it in 2 mL distilled water and centrifuged as above. After the third wash the pellet was resuspended in 2 mL of methanol and vortexed for about 15 s, incubated for 30 min at room temperature, vortexed and centrifuged as above. The concentration of chlorophyll in the supernatant was determined by measuring absorbance at 665 nm and the chlorophyll content was calculated as follows.


$$\mu g\;Chlorophyll/ml=\frac{13.43\;A665v}{lV}$$

Where A665 is absorbance at 665 nm, *v* is the volume of solvent used (ml), l is the spectrophotometric cell length (cm) and *V* is the sample volume (ml).

### 2.6 Extraction and measurement of phycocyanin

To extract phycocyanin 10 mL culture was centrifuged at 4000 rpm for 5 min, the pellet washed twice using distilled water, resuspended in 5 mL of 0.1 M (pH7) phosphate buffer and kept at −20 °C overnight and then subjected to five freezing and thawing cycles. The sample was thawed and subjected to sonication for 30 min at room temperature (Model 704, Scientech Ultrasonic Cleaner, South Africa). After centrifugation for 10 min at 4000 rpm, phycocyanin (C-PC) content was determined from the supernatant by measuring absorbance at 615 nm and 652 nm. Phycocyanin concentration was determined as shown below (25).


$$C-PC\left(g.L^{-1}\right)=\frac{A_{615}-0.474\left(A_{652}\right)}{5.34}$$

Where *A*_615_ is absorbance at 615 nm and A_652_ is the absorbance at 652 nm.

### 2.7 pH measurement

The pH of the culture media was measured at regular intervals throughout the cultivation period to detect if there are changes associated with the growth of *Spirulina* CH22. pH measurements were performed using a calibrated digital pH meter. The pH of the different media (medium T1, medium T7 and Zarrouk’s medium) were adjusted to 10.5 to 10.7 using sodium carbonate and inoculated with the appropriate *Spirulina* culture. During cultivation no pH adjustment was made and measurements were taken over the growth period in the different media.

### 2.8 Statistical analysis

Statistical analysis was performed to evaluate growth kinetics, biomass productivity, chlorophyll, and phycocyanin content. All experiments were conducted in triplicates for each treatment. Results are presented as mean ± standard deviation. Differences among treatments were assessed using one-way analysis of variance (ANOVA) implemented in GenStat software (18th edition). The mean separation was performed using Fisher’s Protected Least Significant Difference (LSD) test at a 95% confidence level. Prior to analysis, data were tested for normality and homogeneity of variances using the Shapiro–Wilk test and Bartlett’s tests, respectively.

## 3 Results

### 3.1 Isolation and identification the strain

Isolate CH22 was obtained from Lake Chitu, Ethiopia, a soda lake found in the African Rift Valley. Soda lakes of the Africa Rift Valley are unique habitats characterized by high primary production, high salinity, and alkalinity (36, 37). In Lake Chitu *Spirulina* is the dominant microalgae, often forming a thick layer of green biomass (38). Based on sequence similarities of the16S rRNA gene and the phycocyanin *cpcA* and *cpcB* genes, isolate CH22 was identified as a strain of *Limnospira platensis* (formerly known as *Spirulina platensis* or *Arthrospira platensis*). The sequence data was deposited at GenBank with an accession number of PP905718.

### 3.2 Optimum NPK fertilizer concentration for growth

**Figure 1:**
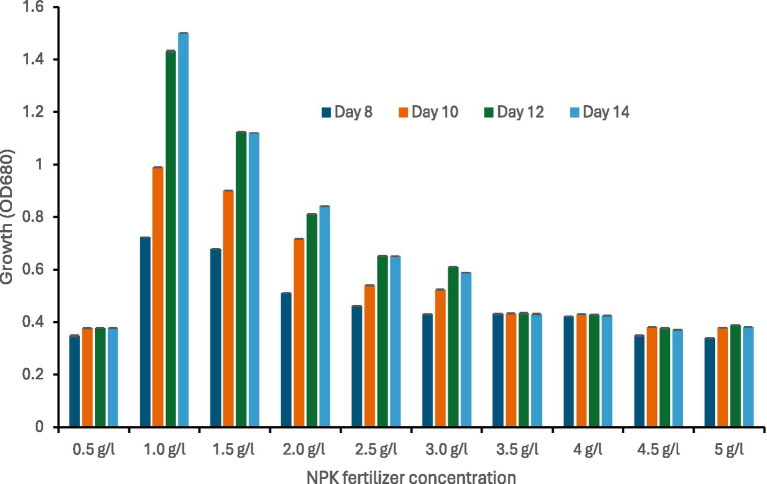
Growth of *Spirulina* CH22 in the presence of different concentrations of NPK fertilizer. Growth was measured by measuring OD680 after the 8th, 10th, 12th, and 14th of cultivations. Values presented as mean ± standard deviation (*n* = 3).

**Figure 2:**
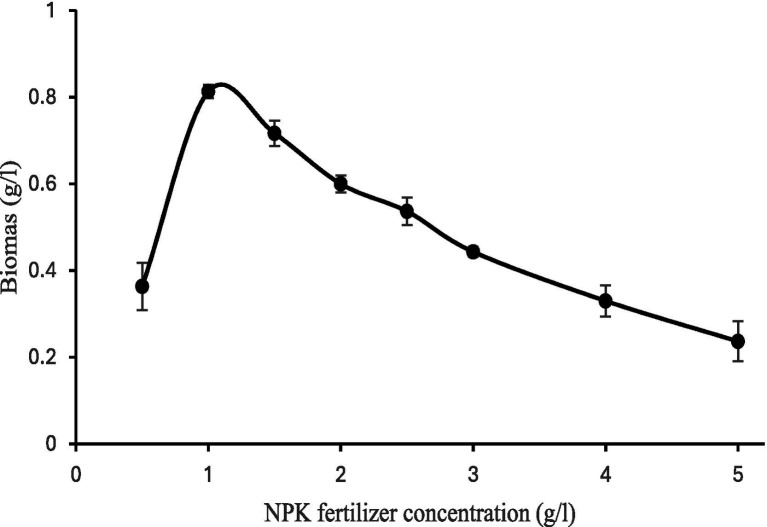
Biomass accumulation of *Spirulina* CH22 grown in different concentrations of NPK fertilizer. Values are presented as mean ± standard deviation (*n* = 3).

When *Spirulina* CH22 was grown in the presence of different concentrations of NPK fertilizer the highest growth was observed in the presence of 1.0 g/L. As concentration increased growth decreased (Figure 1). The manufacturer of the NPK fertilizer declared a total nitrogen content of 62.9 g nitrogen per kilogram. Therefore, based on the declared nitrogen content we calculated that to achieve the same nitrogen content as the nitrate used in Zarrouk’s medium, up to 6.5 g/L NPK fertilizer is required. However, at a fertilizer concentration of above 1.5 g/L growth was significantly retarded (Figures 1, 2). In addition, maximum biomass yield was measured when of *Spirulina* CH22 was grown using 1 g/L fertilizer (Figure 2). Biomass yield then decreased with further increase in fertilizer concentration.

### 3.3 Growth kinetics

**Figure 3:**
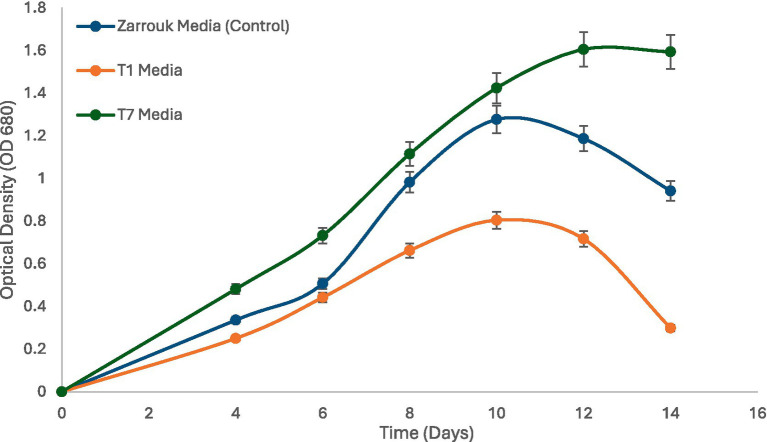
Time course of growth of *Spirulina* CH22 in different media. The media used include standard Zarrouk medium (control) and NPK fertilizer media supplemented with (T7) or without (T1) magnesium, calcium, and iron. Values are presented as mean ± standard deviation (*n* = 3).

Growth kinetics of *Spirulina* CH22 grown in in the different cultivation media was monitored by measuring optical density at 680 nm (OD₆₈₀) at regular intervals. Cultures grown in standard Zarrouk’s medium showed a steady increase in OD₆₈₀ and reached a plateau between days 10 and 12. In medium T1 (NPK fertilizer without mineral supplementation), throughout the cultivation period, growth was consistently lower than Zarrouk’s medium (Figure 3). In contrast, cultures grown in medium T7 (NPK fertilizer supplemented with magnesium, calcium, and iron) exhibited higher growth than Zarrouk’s medium (Figure 3). These results indicate that supplementation fertilizer-based media with iron, magnesium, and calcium lead to a better growth performance.

The specific growth rate and doubling time of cells grown in the three media also differed with medium T7 giving the highest specific growth rate and the shortest doubling time than Zarrouks (Table 2). The lowest specific growth rate and the highest doubling time was recorded for cells grown using medium T1.

**Table 2:** Specific growth rates and doubling times of *Spirulina* CH22 grown in different media.

Treatments/media	Specific growth rate (OD/day)	Doubling time (t*d*)
Zarrouk’s medium	0.2676 ± 0.002^b^	2.590236 ± 0.02^b^
Medium T1	0.243 ± 0.002^a^	2.852458 ± 0.03^a^
Medium T7	0.3215 ± 0.001^c^	2.155979 ± 0.01^c^

The specific growth rate of medium T7 was significantly higher than that of Zarrouk’s medium and medium T1. Values are presented as mean ± standard deviation (*n* = 3), and different superscript letters indicate statistically significant differences between treatments at *p* < 0.05.

### 3.4 Biomass yield and protein content

**Figure 4:**
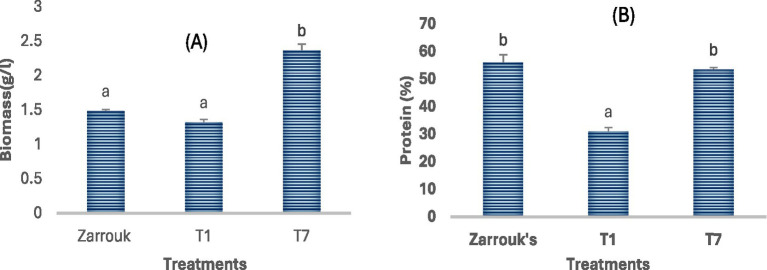
Biomass **(A)** and protein **(B)** content of *Spirulina* CH22 cultivated in different media. The growth media included standard Zarrouk’s medium (Zarrouk’s) and NPK fertilizer without (T1) or with (T7) magnesium, calcium, and iron supplementation. Values are presented as mean ± standard deviation (*n* = 3). Different lower-case letters indicate statistically significant differences among treatments (*p* < 0.05).

Biomass yield of *Spirulina* CH22 grown in the three different media was determined by measuring dry weight of cells harvested after the end of the exponential phase. Biomass yield of cells grown in medium T7 showed a 31% increase in biomass yield over Zarrouk’s medium. A significantly lower biomass yield was obtained for cells grown in medium T1 (Figure 4A).

On the other hand, no difference in protein content of the biomass was observed for Spirulina CH22 grown in medium T7 and Zarrouk medium. Thus, the protein content of *Spirulina* CH22 biomass grown in medium T7 and Zarrouks medium were 54 and 56%, respectively (Figure 4B).

### 3.5 Phycocyanin and chlorophyll content

**Figure 5:**
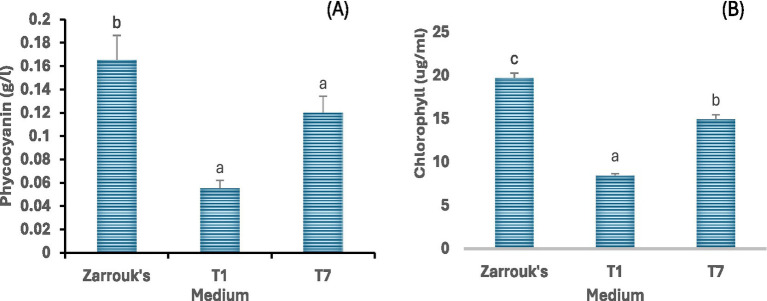
Phycocyanin **(A)** and chlorophyll **(B)** content of *Spirulina* CH22 grown in different media. The growth media included standard Zarrouk’s medium (Zarrouk’s) and NPK fertilizer without (T1) or with (T7) supplementation. Values are presented as mean ± standard deviation (*n* = 3). Different lower-case letters indicate statistically significant differences among treatments (p < 0.05).

Pigment production was influenced by cultivation medium used. Phycocyanin content was highest in cultures grown in Zarrouk’s medium, followed by those grown in medium T7. On the other hand, cells grown in medium T1 produced markedly lower phycocyanin (Figure 5A). Similarly, the amount of chlorophyl produced in Zarrouk’s medium and higher than medium T7 and the chlorophyll content cells grown in medium T1 was the lowest of the three media tested (Figure 5B).

### 3.6 Change in culture medium pH during cultivation

**Figure 6:**
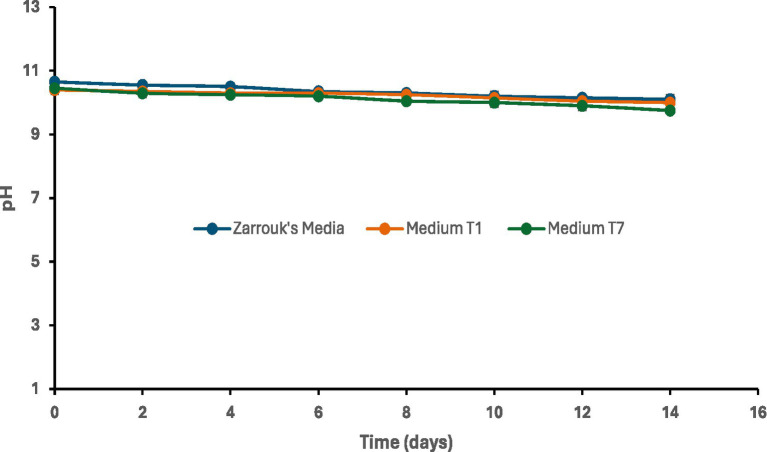
pH change of the different media inoculated with *Spirulina* CH22.

The initial pH of all the media used was in the range 10.5 to 10.7 and remained in the alkaline range throughout the cultivation period (Figure 6). After 12 days if cultivation the pH slightly dropped from about 10.5 to 10.1.

### 3.7 Cost analysis of cultivation media

Cost analysis based on current media component market prices in Botswana revealed substantial differences among cultivation media (Table 3). In Zarrouk’s medium, sodium nitrate, potassium phosphate, and potassium sulfate together accounted for approximately 72% of the total medium cost, with sodium nitrate alone contributing 48%. Replacement of these components with NPK fertilizer reduced the overall medium cost by approximately 68% in medium T1 and 63% in medium T7. Importantly, the optimized fertilizer-based medium (T7) achieved this cost reduction while supporting higher biomass yield and maintaining protein content comparable to that obtained in Zarrouk’s medium.

**Table 3:** Cost of the different medium components used for *Spirulina* cultivation and contribution to the overall medium cost based on current price in Botswana.

Medium component (g/l)	Amount added (kg/m^3^ medium)	Zarrouk’s medium		Medium T1		Medium T7	
		Cost per m^3^ (BWP)	Contribution (%)	Cost per m^3^ (BWP)	Contribution (%)	Cost per m^3^ (BWP)	Contribution (%)
NaNO_3_	2.50	151.4	48.29	–	–	–	–
K_2_HPO_4_	0.50	42.6	13.6	–	–	–	–
K_2_SO_4_	1.00	30.7	9.78	–	–	–	–
MgSO_4_.7H_2_O	0.20	4.3	1.38	–	–	4.31	3.76
CaCl_2_.2H_2_O	0.04	1.1	0.34	–	–	1.07	0.93
FeSO_4_.7H_2_0	0.01	0.1	0.02	–	–	0.08	0.07
EDTA	0.08	9.4	2.99	–	–	9.39	8.18
Na_2_CO_3_	10.00	37.8	12.06	37.8	37.82	37.8	32.93
NaCl	1.00	16.8	5.35	16.76	16.77	16.76	14.6
NPK (2:3:2)	1.00	–	–	26	26.02	26	22.65
Trace metals	1 liter	19.4	6.18	19.38	19.39	19.38	16.89
TOTAL	–	313.5	100%	99.94	100%	114.78	100%

## 4 Discussion

### 4.1 Growth in low cost media

The availability of non-arable land, combined with high solar irradiance throughout the year make the semi-arid regions of Southern Africa highly favorable for commercial scale *Spirulina* cultivation. However, high media cost is a major bottleneck that render the final product expensive for most users. Therefore, to use the full potential of *Spirulina* as food and animal feed supplement in the region there is a need to lower the production cost by reducing media cost and make it affordable for most users, especially for people living in resource limited regions. In commercial scale *Spirulina* cultivation the costs of labor and growth medium are the two major contributors of the high production cost (25, 26). In the Southern Africa region where youth unemployment is a major challenge, cost and availability of labor may not be a major challenge. On the other hand, cost and access to the components of the standard nutrient media could be a major challenge limiting large commercial scale *Spirulina* cultivation. Zarrouk’s medium, which is the standard medium used for the cultivation of *Spirulina* contains NaNO_3_, K_2_HPO_4_, and K_2_SO_4_ as sources of nitrogen, phosphorus, and sulfur, respectively (27, 28). When calculated based on the current cost of medium ingredients in Botswana, the above three components accounted for about 72% of the total cost of the growth medium and NaNO_3_ alone accounted for 48% of the total medium cost (Table 3). Different studies reported replacement of the nitrate in Zarrouk’s medium with low-cost nitrogen sources such as urea, ammonium salts, fertilizer, and nitrogen rich organic wastes leading to a significant reduction in *Spirulina* production cost (41–44). However, the use of these alternative nitrogen sources requires careful optimization as growth and synthesis of pigments such as phycobiliproteins are highly influenced by the composition of the growth medium (23). Excess nitrogen, particularly from sources such as urea which can degrade into ammonia, may lead to cellular damage and inhibit pigment formation (44).

*Spirulina* CH22 used in this study was able to grow well and give good biomass yield using NPK fertilizer as a low-cost cultivation media. Since the strain is newly isolated from a naturally occurring alkaline habitat, it was able to grow at high alkaline pH. Moreover, the pH did not show any drastic change during the entire cultivation period (Figure 6). This is an important property because it helps to limit growth of other contaminant microalgae, especially when in open pond cultivation. As a newly isolated strain, before large scale cultivation and large-scale use there is probably a need to determine its safety for use as animal feed supplement or for other applications. However, it is also worth noting that the medium developed in this study is a general medium which could be used for most know *Spirulina* strains (23). Currently, both the U. S. Food and Drug Administration (FDA), Food and Agriculture Organisation (FAO) and the European Food Safety Agency (EFSA) has approved and recognized *Spirulina* as safe for food and feed applications (6, 12, 16, 23). Based on the laboratory scale cultivation of *Spirulina* observed in this study, the use of medium T1 and medium T7 resulted in a 68 and 63% reduction in the cost of cultivation medium, respectively (Table 3). Therefore, by extrapolation to large scale production, the observed reduction in the cost of growth medium could lead to a significant reduction in the production cost of *Spirulina*. Furthermore, the ready availability of NPK fertilizer in the local market could encourage *Spirulina* cultivation in the Southern Africa region and in turn its availability could encourage people to use it for different applications. This in turn could have a positive impact on the region’s economy through job creation and promotion of sustainable livelihoods (24, 33). For example, with some technical support, people in rural and semiurban areas could start *Spirulina* production and gain economic benefits. *Spirulina* biomass can also serve as a source of high-value compounds such as phycocyanin, which has important applications in the food and cosmetic industries. For example, a financial model study carried out in South Africa showed that local investment in *Spirulina* cultivation can reduce the production cost of phycocyanin, thereby potentially contributing to the country’s bioeconomy (33).

When *Spirulina* CH22 was grown in liquid medium containing NPK fertilizer supplemented with sodium chloride, sodium carbonate, and sodium bicarbonate (medium T1), maximum growth and biomass yield was observed at a concentration of 1 g/L. As concentration increases above 1 g/L, a decline in growth was observed (Figures 1, 2). Ammonium salts and urea are the most common nitrogen compounds used in the formulation of NPK fertilizer (41, 45). Although *Spirulina* is capable of assimilating ammonia, concentration increases above a critical value could cause disruption of photosynthesis and oxidative stress leading to a reduction in biomass and pigment production (42). For example in a different study, during carbon dioxide–assisted cultivation of *Spirulina* optimum growth was reported in the presence of 0.36 and 1.26 g/L fertilizer and at concentrations above 1.5 g/L growth started to declined (46). Therefore, to achieve higher *Spirulina* biomass yield, careful optimization and continuous monitoring of the fertilizer concentration could be of critical importance.

Although *Spirulina* CH22 grows using medium T1 its growth was lower than Zarrouk medium (Table 2). However, upon supplementation of medium T1 with magnesium, calcium, and iron, at concentrations normally used in the standard medium, a better growth of *Spirulina* CH22 was achieved (Figure 3). Thus, *Spirulina* CH22 grown using medium T7 showed higher specific growth rate and a shorter doubling time than medium T1 and Zarrouk’s medium (Table 2). NPK fertilizer is commonly used to supply nitrogen, phosphorus, and potassium for plant growth and the other metal ion requirements are obtained from the soil. Iron, magnesium, and calcium have crucial functions in photosynthesis, biomass yield, and in maintaining metabolic homeostasis (47). Therefore, when *Spirulina* is grown using NPK fertilizer, to achieve higher biomass yield, it is important to supplement the medium with metal ions that are essential for growth and metabolism.

### 4.2 Biomass and protein content of *Spirulina* CH22 grown in different media

Due to its high protein content which ranges from 55 to 70% on dry weight basis and a good balance of amino acids, *Spirulina* is considered ideal for food and animal feed applications (12, 13, 48, 49). Biomass yield and protein content of *Spirulina* is known to be influenced by the genetic makeup of the strain, the composition of the growth medium, and the environmental conditions during cultivation such as pH, salinity, and light intensity (49–52). Compared with Zarrouk medium, *Spirulina* CH 22 grown using medium T7 showed a 31% increase in biomass yield (Figure 4A). But when grown in medium T1 biomass yield significantly decreased (Figure 4A). The biomass yield of *Spirulina* CH22 grown using medium T7 was also higher than the biomass yield of another *Spirulina* strain grown using NPK fertilizer (46). The increase in biomass yield observed in medium T7 compared to NPK fertilizer alone (medium T1) could be a result of the supplemental nutrients added, for example iron. Previous studies showed that moderate supplementation of iron increases the synthesis of carbohydrates and lipids and this results in high biomass yield (45). This shows that medium composition plays a key role in determining the biomass yield of *Spirulina.*

Compared to the standard medium, when *Spirulina* CH22 is grown using plain NPK fertilizer (medium T1) its protein content decreased by 25% (from 56 to 31%). However, up on supplementation of the NPK fertilizer medium with metal ions (medium T7) protein content increased to 54% almost the same as the standard medium (Figure 4B). Both iron and magnesium are important components of photosynthetic pigments which boosts protein synthesis. In addition, magnesium is a component of ribosomes and supports protein synthesis (53). However, despite a 31% increase in biomass yield over the standard medium, protein content of cells grown in medium T7 remained unchanged. This could be a result of the genetic make of the strain or the nitrogen supply from the NPK fertilizer at optimum addition without toxicity may be differently assimilated by the organism (54). In addition, other studies have demonstrated the possibility of nitrogen depletion in the media which may also lead to decreased protein synthesis (55). Although the nitrogen supply may not be sufficient, addition of more NPK fertilizer beyond a critical concentration could lead to ammonia toxicity. To overcome such challenges, one potentially useful approach to increase protein synthesis while minimizing ammonia toxicity could be to use fed batch culture where the fertilizer could be added sequentially under controlled condition (44). On the other hand, low supply of nitrogen could result in a decrease in protein content while increasing other cellular components such as carbohydrate and lipid synthesis (55).

The ability of *Spirulina* CH22 to grow using NPK fertilizer supplemented with selected mineral salt solutions could allow to cut production cost by up to 63% (Table 3) while biomass yield increased by 31% and protein content remained almost unchanged (56% vs. 54%). The mineral salts added to the fertilizer-based medium (medium T7) have only small contribution to the overall cost of the cultivation medium allowing the fertilizer-based formulation to remain substantially cheaper than the conventional Zarrouk’s medium. In the process of production of *Spirulina*, the high cost of the medium components used to formulate Zarrouk’s medium significantly increases the production cost and make the final product expensive. For example, to use *Spirulina* as animal feed additive the final product must be affordable to farmers. If the production cost is high, *Spirulina* will remain inaccessible to most farmers, especially in developing countries.

### 4.3 Phycocyanin and chlorophyll production

Phycocyanin is a blue pigment containing protein and non-protein components which acts as a secondary pigment assisting in the process of photosynthesis (56). In recent years phycocyanin has attracted considerable attention because of its application in the food, cosmetic and pharmaceutical industries (52–58). Therefore, because of its high market demand, in recent years there is a growing interest to develop cost effective methods for its production (33, 59, 60).

*Spirulina* CH22 produced the highest amount of phycocyanin when grown in Zarrouk medium but productivity dropped by more than 90% when grown in medium T1 (Figure 4A). However, when medium T1 was supplemented with Mg, Ca, and Fe (medium T7), phycocyanin production more than doubled. These metal ions are important in photosynthesis (iron and magnesium) and for the stabilization of the cell wall and support better growth and pigment synthesis (45). However, compared to Zarrouk’s medium, less phycocyanin was produced in medium T7 which probably indicates the need to optimize the medium composition, especially in terms of the nitrogen supply. Since NPK fertilizer contain ammonium salts which under alkaline pH convert to ammonia, there is need to use the right concentration to avoid ammonia toxicity due to high concentration and nitrogen deficiency due to low concentration, both of which known to affect phycocyanin production (42, 44). Different studies have also demonstrated the role of the growth medium and environmental conditions in determining phycocyanin production by *Spirulina* (69−71).

The chlorophyll content of *Spirulina* is also known to be influenced by the composition of the medium (19). The amount of chlorophyll produced by *Spirulina* CH22 varied with the cultivation medium with the highest chlorophyll content observed in the standard medium followed by medium T7 (Figure 5A). Chlorophyll content of cells grown in medium T7 was about 1.8 folds higher than in medium T1 which could be a result of supplementation with magnesium and iron. Since magnesium is a component of chlorophyll, its presence in sufficient quantity in the medium regulate pigment biosynthesis (62). Similarly, iron plays a key role in chlorophyll synthesis and its deficiency could lead to chlorosis (63−66). Unlike plants that obtain their iron and magnesium requirements from soil and natural environments (67, 68), when using NPK fertilizer for commercial scale *Spirulina* cultivation it is important to provide the right amount of these metal ions for optimum chlorophyll synthesis (45, 46).

### 4.4 Analysis of pH

The pH of all media (Figure 5) remained within the alkaline range (pH 9.7–10.7) suitable for *Spirulina* cultivation throughout the experimental period, consistent with its preference for alkaline conditions (6, 10). The minor variations observed over time do not indicate any substantial change in pH but rather reflect typical fluctuations that occur in culture systems. These small changes may be associated with the exchange of CO₂ between the culture medium and the surrounding air, as well as metabolic activity during cultivation, including carbon utilization and shifts in carbonate equilibrium within the medium, as *Spirulina* assimilates inorganic carbon sources such as CO₂ and bicarbonate during growth (65). Overall, the stability of pH across all treatments indicates that both the fertilizer-based media and the control maintained conditions conducive to *Spirulina* growth.

## 5 Conclusion

The environmental conditions prevailing in semi-arid regions of Southern Africa are favorable for the cultivation of *Spirulina* under controlled systems. In this study, *Spirulina* CH22 was isolated from a soda lake and successfully cultivated at laboratory scale using a low-cost NPK fertilizer-based medium supplemented with essential metal ions. Compared with the standard medium, the supplemented fertilizer-based medium (T7) supported a 31% increase in biomass yield while maintaining comparable protein content of 54%. In addition, the use of fertilizer-based media reduced medium cost by approximately 63%. These findings demonstrate that fertilizer-based media formulated from locally available agricultural inputs can support *Spirulina* cultivation while reducing medium formulation costs. The comparable protein content and improved biomass yield observed in T7 relative to the control indicate that nutrient supplementation can enhance growth performance without compromising biomass quality. The results of this study provide a basis for further investigation into the development of low-cost *Spirulina* cultivation systems in resource-limited settings, such as in the semi-arid regions of Southern Africa. In addition, the biomass produced under these conditions may have potential for animal feed applications after further nutritional evaluations and feeding trials.

## Data Availability

The genetic sequence referred during the current study is available in the NCBI (National Center for Biotechnology Information) repository and assigned Accession Number; PP905718. GeneBank Link; https://www.ncbi.nlm.nih.gov/nucleotide/PP905718.1?report=genbank&log$=nuclalign&blast_rank=1&RID=J83BU6T3014&from=1&to=1031.
